# Biointegrated Multilayer Stretchable OLED Platform With Strain‐Decoupled Architecture for Durable Phototherapeutic Applications

**DOI:** 10.1002/advs.76176

**Published:** 2026-06-16

**Authors:** Young Hyun Son, Myeongheon Lee, Jun‐Yeop Song, Hyo‐Jung Kwon, Kyung Cheol Choi, Jeong Hyun Kwon

**Affiliations:** ^1^ School of Electrical Engineering Korea Advanced Institute of Science and Technology (KAIST) Daejeon Republic of Korea; ^2^ School of Semiconductor Engineering Chungbuk National University Cheongju Chungcheongbuk‐do Republic of Korea; ^3^ College of Veterinary Medicine Chungnam National University Daejeon Republic of Korea

**Keywords:** bio medical application, multifunctional encapsulation, multilayer architecture, resolution–stretchability trade‐off, stretchable oleds

## Abstract

To advance wearable medical technology beyond its current limits, stretchable organic light‐emitting diode (SOLED) displays must become practical. Achieving this requires both mechanical softness and durable environmental protection, yet conventional SOLED platforms are limited by a resolution–stretchability trade‐off and the fracture‐prone nature of inorganic encapsulation layers. Here we report a multilayer SOLED architecture that separates light emission and mechanical deformation into vertically stacked planes. By decoupling emissive pixel islands from deformable interconnects, the design enables high‐fill‐factor pixel patterning while improving mechanical compliance and structural reliability. Finite‐element analysis and experimental measurements show that the multilayer stack redistributes tensile strain into compliant elastomeric layers, delivering approximately 54% system‐level stretchability. Stable electroluminescence is maintained under repeated loading and 50% uniaxial tensile strain. For outdoor and long‐term operation, we further develop a hybrid encapsulation combining atomic‐layer‐deposited nanolaminate distributed Bragg reflector layers with parylene‐C. The resulting barrier exhibits a barrier performance of 1.26 × 10^−^^6^ g m^−^^2^ day^−^^1^, achieves 99.87% ultraviolet blocking, and preserves device operation under cyclic deformation and hygrothermal stress. In a murine wound‐healing model, the SOLED patch conformally covered skin and accelerated healing by over 50% compared with controls, demonstrating its strong potential for wearable therapeutic applications.

## Graphical abstract

A multilayer stretchable organic light‐emitting diode (SOLED) patch enables conformal, high‐fill‐factor phototherapeutic illumination through vertically decoupled electrodes and robust encapsulation. The textile‐based SOLED platform delivers stable skin‐contact red light for wearable phototherapy and 5‐aminolevulinic acid (ALA)‐mediated photodynamic treatment, accelerating in vivo wound closure through enhanced keratinocyte proliferation, angiogenesis, collagen deposition, and TGF‐β/SMAD signaling.

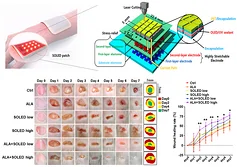

## 1 Introduction

Recent advances in wearable biomedical systems have increased the demand for soft and conformal light‐delivery platforms for phototherapeutic applications such as photobiomodulation and photodynamic therapy (PDT) [1, 2, 3, 4, 5, 6, 7]. Despite significant progress in wearable and implantable phototherapeutic devices, current light‐delivery systems still face critical limitations including poor conformal skin contact, nonuniform light distribution, limited mechanical durability, and unstable operation under repeated deformation and humid environments [4, 5, 6, 7, 8]. In particular, conventional rigid or semi‐flexible light sources often exhibit insufficient mechanical compliance to dynamically deformable biological tissues, resulting in unstable optical coupling and reduced therapeutic effectiveness. Therefore, the development of mechanically reliable and uniformly emissive wearable phototherapeutic platforms remains an important challenge for practical biointegrated healthcare systems [8, 9, 10, 11, 12].

Among various light‐emitting technologies, organic light‐emitting diodes (OLEDs) have emerged as highly promising candidates for wearable phototherapy owing to their intrinsic mechanical softness, ultrathin form factor, spectral tunability, low‐voltage operation, and capability for large‐area surface emission [12, 13, 14]. Unlike conventional inorganic LEDs or laser‐based systems, OLEDs can provide highly uniform and gentle surface illumination while maintaining excellent mechanical flexibility and conformability to curved biological surfaces [15, 16, 17, 18]. Nevertheless, the realization of stretchable OLED (SOLED) platforms suitable for practical biomedical applications remains highly challenging because simultaneous achievement of high fill factor, mechanical stretchability, environmental stability, and reliable encapsulation is difficult [19, 20, 21]. In particular, conventional structurally engineered SOLEDs based on island–bridge geometries inevitably suffer from a trade‐off between emissive area density and strain accommodation capability, limiting their applicability for uniform phototherapeutic light delivery under dynamic deformation conditions [22, 23].

To realize mechanically reliable wearable OLED systems, thin‐film encapsulation (TFE) technologies have been extensively investigated as essential components for protecting organic optoelectronic devices from oxygen, moisture, ultraviolet (UV) irradiation, and thermal degradation. In particular, atomic layer deposition (ALD)‐based inorganic barrier layers have attracted significant attention owing to their excellent conformality, nanoscale thickness controllability, and extremely low water vapor transmission rates (WVTRs) [24, 25, 26, 27]. Recent advances in multilayer TFE structures integrating inorganic nanolaminates, organic interlayers, and distributed Bragg reflector (DBR)‐based multifunctional barriers have demonstrated improved environmental stability and optical functionality for wearable OLED applications [28, 29]. Nevertheless, conventional inorganic ALD barriers such as Al_2_O_3_ still suffer from intrinsic brittleness and mechanical degradation under repeated deformation, thermal stress, and humid operating environments, leading to crack formation, interfacial failure, and deterioration of long‐term barrier reliability [30, 31]. Therefore, the development of mechanically robust and environmentally stable encapsulation structures remains a critical prerequisite for practical biointegrated OLED systems.

In parallel, various approaches have been explored to realize SOLED systems, including elastomer‐supported planar OLED fabrication, transfer printing of micro‐emitters onto stretchable substrates, and structurally engineered island–bridge architectures incorporating rigid emissive regions interconnected by deformable electrodes [32, 33, 34, 35]. Among these strategies, structural approaches are considered particularly attractive because they can preserve established OLED fabrication processes while enabling mechanical stretchability at the system level [36, 37, 38, 39]. However, conventional island–bridge SOLED architectures inevitably suffer from trade‐offs between mechanical deformability and emissive area density because increased stretchability generally requires larger non‐emissive interconnection regions (Table S1) [40, 41, 42]. Such limitations become more critical in wearable phototherapeutic applications, where large‐area uniform light delivery, stable skin conformity, and high fill factor are essential for maintaining therapeutic effectiveness. These unresolved challenges motivate the development of advanced SOLED architectures capable of simultaneously achieving high mechanical reliability, high fill factor, environmental stability, and uniform surface illumination under dynamic deformation conditions.

To address these challenges, we propose a biointegrated multilayer SOLED platform featuring vertically decoupled emissive and interconnection architectures combined with multifunctional hybrid encapsulation. By spatially separating emissive pixels and deformable interconnects into different mechanical planes, the proposed architecture simultaneously achieves high fill factor, stable stretchability, and mechanically reliable operation under tensile deformation. In addition, the incorporation of multifunctional encapsulation structures enhances environmental reliability while maintaining optical and mechanical stability for wearable phototherapeutic applications. As a result, the developed SOLED platform provides highly uniform surface illumination, stable conformal skin integration, and durable operation under mechanically dynamic and humid biomedical environments, thereby offering a promising strategy for next‐generation wearable phototherapeutic systems.

## 2 Results and Discussion

### 2.1 Integrated Stretchable OLED Platform for Wearable Applications

Wearable electronic systems require mechanically compliant device platforms capable of conformal integration with soft and dynamically deformable biological surfaces. Conventional flexible electronics provide bendability; however, their limited stretchability restricts stable operation under large mechanical deformation encountered in real‐world wearable environments. Therefore, the development of mechanically robust stretchable platforms capable of maintaining device functionality under tensile strain remains a critical challenge.

**FIGURE 1:**
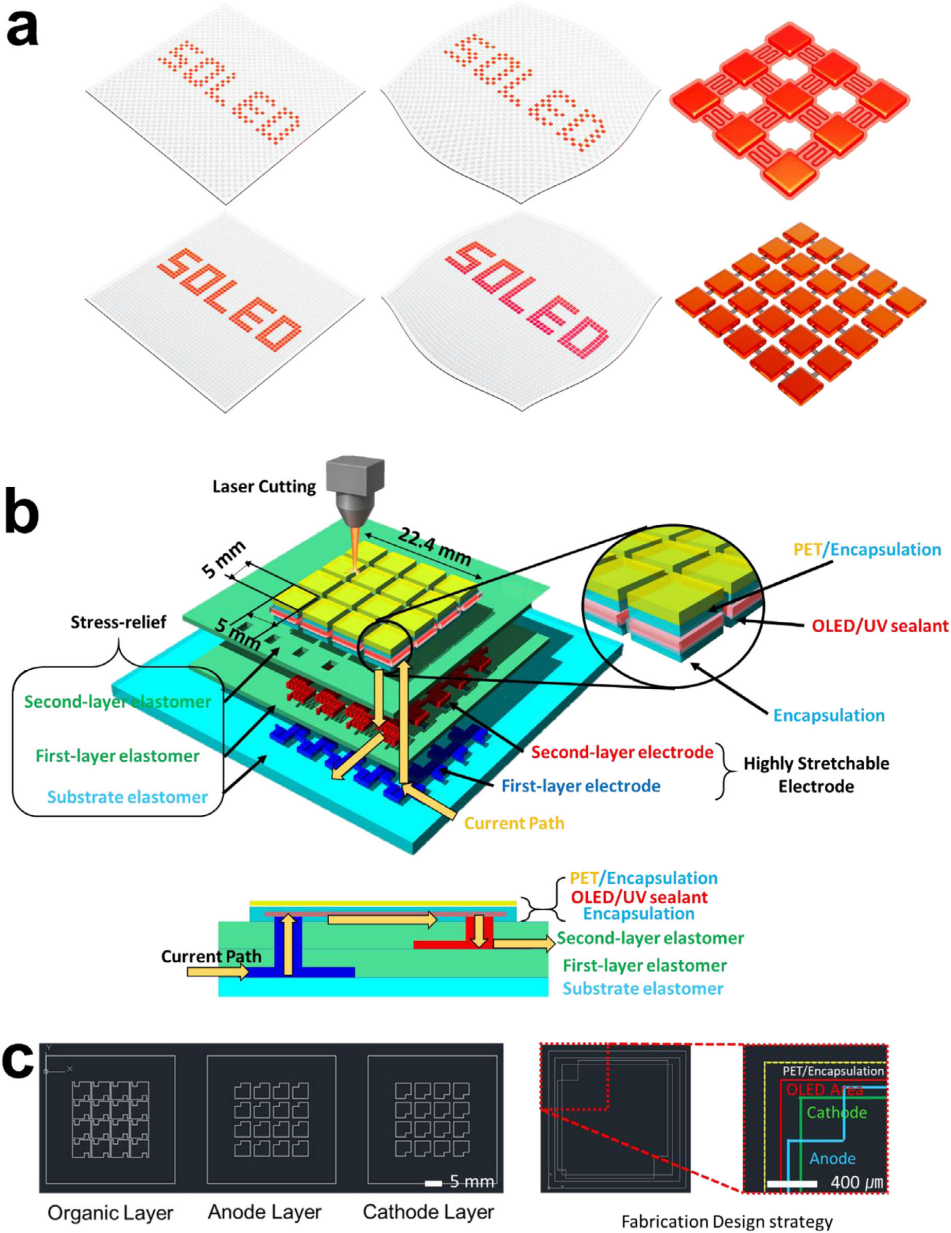
Structural design and mechanical architecture of the triple‐layer stretchable OLED system. (a) Comparison of pixel geometry, strain‐induced deformation behavior, and island–bridge configurations in conventional and triple‐layer stretchable OLED architectures. Schematic illustrations depict the pixel geometry and mechanical response under tensile strain for both structures, together with their corresponding top‐view island–bridge layouts. In the triple‐layer architecture, the island–bridge configuration is embedded beneath the multilayer stack, allowing strain redistribution away from the emissive pixels and thereby enhancing mechanical stability. (b) Exploded isometric view of the integrated device architecture. The system comprises (from top to bottom) a laser‐cut PET frame, thin‐film encapsulation layer, OLED island layer, SU‐8 structural island, vertically separated electrode layers interconnected through via, and an elastomeric substrate. The functional grouping emphasizes the separation of environmental protection layers, emissive components, and strain‐isolating mechanical structures. (c) Design layout of the laser‐cut PET encapsulation framework and the fabrication alignment strategy.

Figure 1a illustrates the structural transition from a conventional single‐layer island–bridge stretchable display to the proposed multilayer architecture. The schematic depicts the undeformed and stretched states of a traditional configuration, where rigid emissive islands are interconnected by deformable bridges. Under applied strain, deformation is primarily accommodated by elongation of the interconnection regions, leading to increased pixel spacing and a reduced fill factor (FF), which can degrade image fidelity. In contrast, the proposed multilayer structure spatially separates emissive islands and electrode interconnections into vertically decoupled layers. The emissive pixels are densely arranged in the upper layer, while the wiring is routed beneath them in a dedicated interconnection plane. This vertical separation enables compact pixel packing and high FF while providing sufficient area for mechanical strain accommodation, thereby overcoming the intrinsic resolution–stretchability trade‐off of conventional single‐layer designs. The FF was defined as the ratio of the emissive pixel area to the total device area, and the detailed calculation method is provided in Figure S1. This vertically decoupled configuration fundamentally redefines the strain‐accommodation pathway, breaking the intrinsic coupling between fill factor and mechanical stretchability that constrains conventional single‐layer island–bridge designs.

Figure 1b illustrates the structural integration strategy of the multilayer SOLED platform, where vertically separated functional layers are assembled to achieve both mechanical compliance and optoelectronic stability. The architecture comprises stretchable interconnect networks embedded within elastomeric substrates, densely packed emissive OLED islands positioned on an upper functional plane, and an encapsulated device stack supported by a laser‐patterned PET framework. The vertical decoupling of emissive pixels and deformable interconnections redistributes mechanical strain into compliant elastomer regions while preserving the structural integrity of the active OLED islands. The laser‐defined PET support layer provides precise geometric control of the emission area and enhances mechanical robustness without sacrificing flexibility. This hybrid multilayer configuration simultaneously improves structural stability, environmental durability, and mechanical stretchability required for wearable applications. Furthermore, the combination of stretchable encapsulation and laser‐patterned PET integration offers process scalability, alignment precision, and improved fabrication reliability, enabling stable and reproducible realization of stretchable organic light‐emitting diodes. A cross‐sectional schematic of the multilayer SOLED platform is further illustrated in Figure 1b to clarify the vertically decoupled routing architecture and electrical connection pathway. In the proposed structure, the emissive OLED layer is positioned on the upper functional plane together with the encapsulation and PET support layer, while the stretchable interconnects are embedded within lower elastomeric layers. Electrical connection between the lower routing electrodes and the upper emissive island is achieved through vertically integrated via contacts, enabling current delivery without occupying additional in‐plane pixel area. This hidden routing configuration allows dense emissive pixel arrangement while spatially separating the strain‐accommodating interconnect network from the active OLED region. As a result, mechanical deformation is preferentially redistributed into the compliant lower elastomeric layers, thereby minimizing effective strain transfer to the emissive stack and maintaining high fill factor under tensile deformation.

Figure 1c illustrates the fabrication strategy integrating laser‐patterned PET with aligned deposition masking for multilayer OLED realization. The PET substrate is precisely laser‐defined to establish the emission window, sealing boundary, and alignment reference, while minimizing thermal or mechanical damage to the active OLED stack. The patterned PET layer serves a dual function: it provides structural reinforcement and simultaneously acts as a process‐aligned masking framework for sequential cathode, anode, and organic layer deposition. This strategy ensures accurate pattern registration, reproducible device geometry, and mechanically stable integration of stretchable OLED devices. By combining laser precision with encapsulation compatibility, the approach enables scalable fabrication without compromising electrical or optical performance during subsequent mechanical processing.

### 2.2 Mechanical Reliability of PET‐Integrated Multilayer Stretchable Platform

**FIGURE 2:**
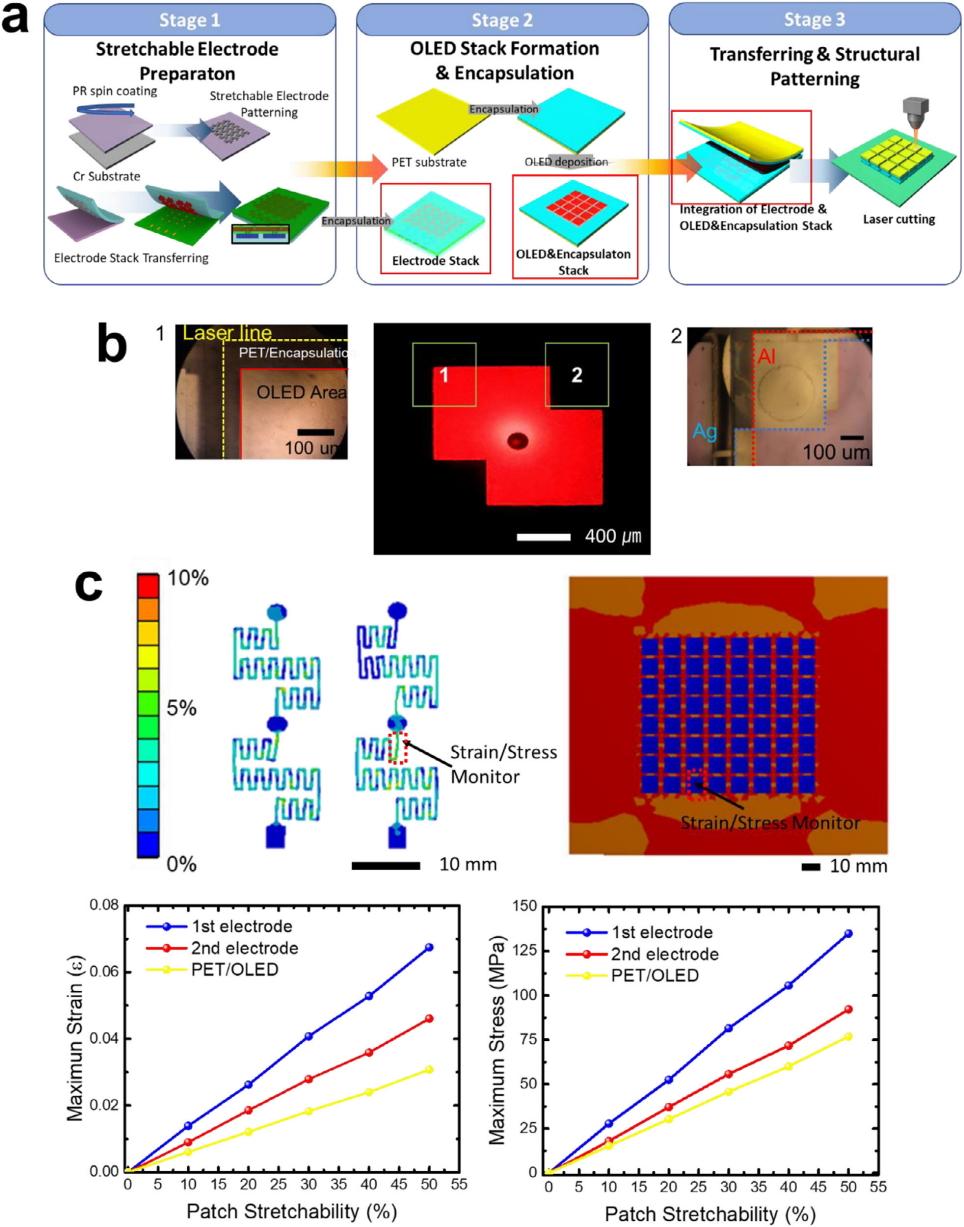
Fabrication strategy and strain‐isolation mechanism of the multilayer SOLED platform. (a) Schematic illustration of the fabrication sequence for the multilayer SOLED platform. The stretchable multilayer substrate and the laser‐cut PET framework are prepared in parallel, followed by thin‐film encapsulation and deterministic lamination to assemble the final integrated device. (b) Alignment strategy between the PET encapsulation frame and the multilayer OLED island array. Marker‐assisted positioning enables precise registration of the emission window with the underlying pixel regions. (c) Simulated strain distribution within the integrated multilayer stack under uniaxial tensile deformation, demonstrating effective strain isolation of the emissive island region after PET encapsulation. Average von Mises stress values were extracted from the highlighted representative regions of each functional layer.

The fabrication process was performed in accordance with the design rules defined in Figure 1c and is summarized in Figure 2a as a three‐stage integration sequence. In Stage 1, stretchable electrode layers were prepared by photolithographic patterning (PR coating and electrode definition) followed by formation of the metal stack and its transfer onto an elastomer substrate, yielding a mechanically compliant interconnect layer with well‐defined routing geometry. In Stage 2, the top‐emitting OLED device stack was formed and encapsulated by sequential deposition of the organic layers and electrodes within a process‐aligned window, producing an OLED‐and‐encapsulation stack that preserves the intended pixel layout. Although OLEDs can in principle be fabricated directly on stretchable multilayer substrates, the realization of such devices typically requires a top‐emitting OLED architecture. In this configuration, the cathode layer, typically Al, must be deposited first. However, it is well known that Al films deposited on polymer or dielectric surfaces often exhibit relatively rough morphologies due to unfavorable growth characteristics [43]. In our study, we observed that the surface roughness of the Al film strongly depends on both the type of heating boat used for thermal evaporation and the deposition rate. The rough morphology of the deposited Al film can also be attributed to the poor wetting characteristics of the underlying parylene‐C film. Owing to its relatively low surface energy and hydrophobic nature, parylene‐C tends to promote island‐type growth of deposited metals, which can lead to increased grain size and surface roughness. When deposited using a pyrolytic boron nitride boat, a 100 nm thick Al film exhibited a peak‐to‐valley roughness of 123.51 nm (Figure S2). Such a highly rough surface is unsuitable for stable OLED operation because it significantly increases the likelihood of electrical shorts. Indeed, the fabricated top‐emitting devices either failed to turn on or exhibited severely degraded optoelectronic characteristics. To overcome this limitation, we adopted an alternative device configuration in which bottom‐emitting OLEDs were first fabricated on a PET substrate and subsequently integrated with the stretchable multilayer substrate through a lamination process. In Stage 3, the pre‐formed electrode layer and the OLED/encapsulation stack were laminated and integrated through a controlled transfer/assembly step, after which laser patterning of the PET framework was applied to define the emission window, sealing boundary, and overall device outline with high positional accuracy.

This sequential integration strategy enables vertical decoupling of the emissive islands and stretchable interconnections while maintaining geometric precision and structural stability. Detailed fabrication procedures and material parameters are provided in the Figure S3. The laser‐patterned PET framework serves both as a mechanical reinforcement layer and as a geometry‐defining element, ensuring accurate realization of the designed layout and reproducible multilayer integration.

The fabricated multilayer platform is shown in Figure 2b. The emission image confirms uniform light output from the defined OLED active region. Enlarged optical views further verify that the laser‐patterned PET encapsulation precisely defines the device boundary and emission window. Clear separation between the OLED area and the electrode routing layers (Ag and Al) demonstrates accurate alignment and high pattern fidelity achieved through the laser‐assisted integration strategy. These results confirm that the designed multilayer layout is reliably implemented without structural distortion or misalignment.

Finite element simulations were performed under 50% uniaxial tensile strain to evaluate the mechanical response of the integrated multilayer structure (Figure 2c). The simulations were conducted using experimentally relevant material parameters, where the Ecoflex elastomer was modeled with a Young's modulus of 0.5 MPa and a Poisson's ratio of 0.49, while the PET support layer was modeled with a Young's modulus of 1.2 GPa and a Poisson's ratio of 0.38. The strain contour maps reveal that mechanical deformation is primarily localized within the serpentine interconnection regions, while the emissive island array experiences significantly reduced strain. Under an applied tensile strain of 50%, the maximum strain in the emissive island region of the SOLED was reduced by approximately 54.4% compared to that in the first electrode layer, quantitatively confirming effective strain decoupling within the multilayer architecture. Detailed finite element modeling parameters and extended stress analyses are provided in the Figure S4. This strain redistribution effect confirms effective mechanical decoupling between the rigid pixel layer and the compliant routing network.

Quantitative analysis of maximum strain and stress as a function of applied patch stretchability further supports this observation. As the global stretchability increases, strain and stress increase gradually but remain concentrated in the designed interconnection regions, minimizing mechanical loading on the emissive islands. It should be noted that the reported platform‐level stretchability corresponds to the global tensile deformation applied to the entire device structure, whereas the encapsulation layer positioned on the emissive island experiences substantially reduced local strain due to the strain‐relief and redistribution effect of the vertically decoupled multilayer architecture. These results demonstrate that the PET‐integrated multilayer architecture effectively redistributes mechanical deformation and preserves structural integrity under applied tensile strain. Collectively, these results establish that the PET‐integrated multilayer architecture does not merely accommodate deformation, but actively redistributes strain away from the emissive islands, thereby enabling mechanically reliable, high–fill‐factor optoelectronic operation under large tensile deformation.

**FIGURE 3:**
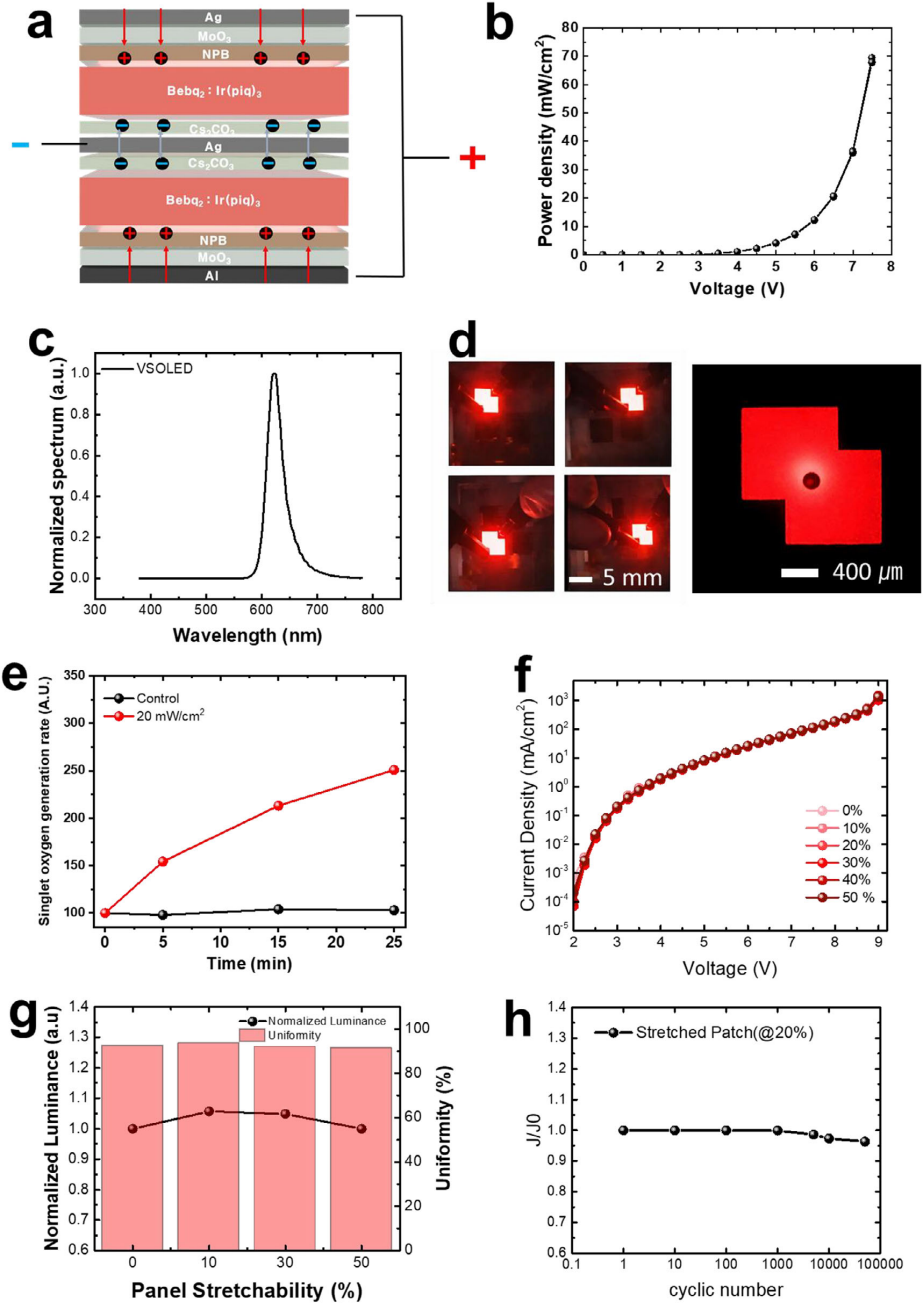
Electro‐optical performance and mechanical stability of the VSOLED‐based SOLED patch. (a) Schematic illustration of the red‐emitting VSOLED device. (b) Power density of the VSOLED. (c) Normalized electroluminescence spectrum of the VSOLED device, exhibiting a well‐defined emission peak in the red region. (d) Photographs of the operating VSOLED device under electrical bias, demonstrating uniform red emission and stable light output across the active area. (e) Singlet oxygen generation rate of the SOLED patch at a power density of 20 mW/cm^2^. (f) Current density–voltage–luminance (*J*–*V*) characteristics of the PET‐integrated device measured under applied tensile strain, demonstrating stable charge injection and light emission behavior during mechanical deformation. (g) Normalized luminance as a function of tensile strain, together with irradiance uniformity measured after 100 stretching cycles at different strain levels. (h) Normalized current density (J/J_0_) as a function of stretching cycle number under repeated tensile loading, demonstrating stable electrical performance and mechanical reliability of the integrated platform.

To achieve high‐power operation, a vertically stacked OLED (VSOLED) architecture comprising two OLED units was developed, enabling a sustained optical output exceeding 20 mW cm^−^^2^ suitable for PDT‐based phototherapy (Figure 3a) [44]. The dual red OLED stack facilitates more efficient current distribution, resulting in improved power density and extended device lifetime. As shown in Figure 3b, the SOLED patch integrated with a VSOLED device is capable of delivering a maximum irradiance of 70 mW/cm^2^. Consequently, when operated at an irradiance of 20 mW/cm^2^ during the in vivo experiments in this study, the device maintained stable performance over prolonged operation. For the PDT test for wound healing, a red VSOLED device was fabricated with its peak emission wavelength tuned to approximately 625–630 nm (Figure 3c). A well‐defined emission peak in the red region is observed, indicating stable radiative recombination within the emissive layer. The spectral profile shows no abnormal broadening or distortion, confirming that the multilayer integration and encapsulation processes do not degrade the intrinsic optical characteristics of the OLED stack.

Figure 3d shows optical images of the operating device under electrical bias. Uniform red emission is observed across the defined active region, demonstrating consistent light output and successful pattern definition. The absence of dark spots or intensity inhomogeneity indicates reliable pixel formation and structural integrity of the multilayer platform.

Singlet oxygen generation upon light irradiation of the photosensitizer using the OLED patch was quantified using a singlet oxygen sensor green (SOSG) assay [44]. In this assay, singlet oxygen reacts to form a fluorescent endoperoxide, characterized by an absorption peak at 504 nm and an emission peak at 525 nm. The results revealed that the rate of singlet oxygen production increased proportionally with both the irradiance and exposure duration of the SOLED patch (Figure 3e). Notably, irradiation at an intensity of 20 mW/cm^2^ for 25 min resulted in a 2.5‐fold increase in singlet oxygen generation compared with the control group.

We additionally evaluated the long‐term operational stability of the SOLED devices under a clinically relevant phototherapeutic operating condition corresponding to an optical power density of 20 mW/cm^2^. The newly added results are presented in the revised Figure S5. The operational lifetime measurements were conducted under continuous operation at 30°C/90% relative humidity (RH), and the luminance evolution of both encapsulated and unencapsulated SOLED devices was monitored over time. The encapsulated SOLED employing the multifunctional multibarrier encapsulation exhibited significantly improved operational stability, with an LT70, defined as the time required for the luminance to decrease to 70% of its initial value, of 127.37 h, whereas the unencapsulated SOLED showed an LT70 of only 18.167 h. These results demonstrate an approximately 7‐fold enhancement in operational lifetime achieved by the multibarrier encapsulation.

The electromechanical stability of the PET‐integrated multilayer SOLED platform was evaluated under applied tensile strain (Figure 3f–h). Despite significant mechanical deformation, the electrical and optical characteristics remain nearly unchanged, demonstrating robust operational stability.

Figure 3f presents the *J*–*V* characteristics of the SOLED device under increasing tensile strain. The current density–voltage curves exhibit negligible variation, demonstrating that the electrical operation of the SOLED is well maintained under mechanical deformation without significant degradation in charge injection or current transport.

Figure 3g shows the normalized luminance variation of the SOLED device under tensile strain and the irradiance uniformity measured after stretching at different strain levels. The normalized luminance remained nearly constant during stretching, indicating that the optical output was well preserved under mechanical deformation. For spatial irradiance analysis, multiple sampling points were evenly distributed across the emitting surface, as shown in Figure S6. The SOLED exhibited an average irradiance of 20.353 mW cm^−^^2^ and a high irradiance uniformity of 95.35%, confirming homogeneous light delivery with minimal spatial intensity variation. After 100 stretching cycles, the irradiance uniformity remained high at 96.24%, 94.76%, and 94.13% under 10%, 30%, and 50% strain, respectively. These results confirm that the SOLED platform maintains stable optical output and spatially uniform light delivery under repeated mechanical deformation, supporting its potential for wearable phototherapeutic applications.

Figure 3h summarizes the normalized current density (J/J_0_) under applied strain, showing only minimal deviation even at elevated tensile levels. This performance stability originates from the vertically separated island–interconnection architecture. By spatially decoupling the emissive islands from the strain‐accommodating interconnection layer, mechanical deformation is redistributed into the compliant lower routing structure, thereby substantially reducing the effective strain experienced by the active OLED region. As a result, structural integrity of the emissive stack is preserved, enabling consistent electrical performance during stretching.

### 2.3 ALD Nanolaminate‐Based Multifunctional Encapsulation

**FIGURE 4:**
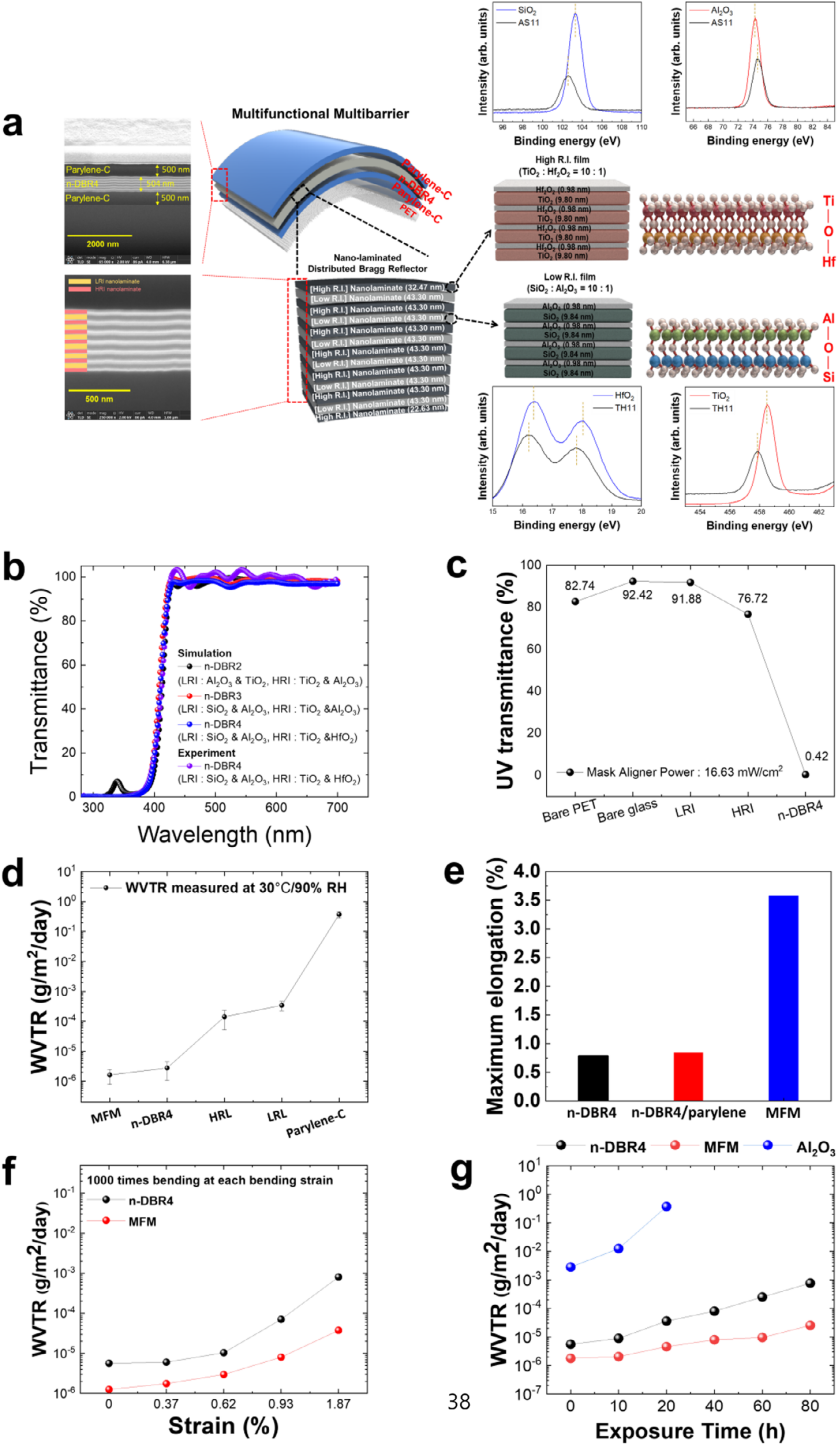
Structure and performance of multifunctional multibarrier encapsulation. (a) Schematic illustration of the MFM encapsulation with a 6.5‐pair n‐DBR structure composed of HRI and LRI nanolaminates. The extended Figure shows a cross‐sectional scanning electron microscope image of the MFM, a schematic diagram, and X‐ray photoelectron spectroscopy spectra indicating Al─O─Si and Ti─O─Hf bonds at the sublayer interfaces. (b) Optical transmittance of different n‐DBR structures obtained from both simulations and experiments. (c) UV transmittance of various substrates and encapsulation films under exposure to 16.63 J/s of UV irradiation. (d) WVTR values of different encapsulation barrier films measured using the Ca corrosion test. (e) Elongation of n‐DBR4, n‐DBR4/parylene‐C, and MFM structures. (f) WVTR changes of n‐DBR4 and MFM films as a function of bending strain. (g) WVTR variations of Al_2_O_3_, n‐DBR4, and MFM films over exposure time at 60°C/90% RH. Data are presented as mean ± standard deviation (*n* = 3).

Thus far, we have discussed the mechanical behaviors of multilayered stretchable substrates for SOLEDs. To achieve SOLEDs that are both mechanically and environmentally reliable, we developed a multifunctional multibarrier (MFM) encapsulation consisting of a parylene‐C/DBR/parylene‐C structure in which the ALD nanolaminate‐based multifunctional DBR serves as the inorganic barrier and parylene‐C as the organic barrier (Figure 4a). For reliable stretchable organic electronic devices for application in outdoor conditions, we developed a multifunctional encapsulation film that could yield a WVTR of 10^−6^ g/m^2^/day and block harmful UV light.

The conventional DBR structure, which is primarily employed in optical fibers and sensors, is multistacked with alternating high‐ and low‐refractive index (HRI and LRI, respectively) layers, with controlled periodic variation and thickness of each layer. We developed the first multifunctional DBR encapsulation system comprising four inorganic materials. In detail, a nanolaminated DBR comprising four inorganic material (n‐DBR4) structures, with each constituent HRI and LRI layer manufactured as a nanolaminate structure, was fabricated for improved WVTR, flexibility, and environmental stability compared with conventional DBR and n‐DBR2 structures. To achieve a mechanically robust, environmentally stable n‐DBR structure that transmitted 95% of the red, green, and blue wavelengths while reflecting 95% of UV wavelengths (< 400 nm), ALD TiO_2_ and HfO_2_ were employed as HRI materials and ALD SiO_2_ and Al_2_O_3_ were used as LRI materials, at a low temperature of 40°C. In the n‐DBR2 structure, the refractive‐index contrast between the two constituent materials generated a photonic stopband spanning approximately 340–400 nm, for which light transmission was suppressed and reflectance was enhanced. As TiO_2_ exhibits intrinsic absorption in the UV region below approximately 380 nm, the transmittance in this range was further reduced. Thus, the low transmittance observed below 400 nm arose from the combined effects of reflection due to the photonic stopband formed by the high/low‐index pair and the TiO_2_ absorption. However, the refractive‐index contrast between Al_2_O_3_ and TiO_2_ is relatively small and, thus, the stopband width was limited. At approximately 340 nm, the TiO_2_ absorption coefficient was not sufficiently high to achieve complete blocking; thus, this absorption did not fully compensate for the reduced reflection. To extend the suppression to the 330–340‐nm range, an n‐DBR with a larger refractive‐index contrast was required. SiO_2_, which has a lower refractive index than Al_2_O_3_, was introduced to form an Al_2_O_3_/SiO_2_ nanolaminate‐based LRI layer. For the HRI, HfO_2_ was incorporated to form a TiO_2_/HfO_2_ nanolaminate high‐index layer. This combination yielded an n‐DBR4 structure with a significantly larger refractive index contrast, in which the HRI layer was a nanolaminate structure with multiple TiO_2_/HfO_2_ periods (TiO_2_‐to‐HfO_2_ sublayer thickness ratio = 10:1) and the LRI layer was a SiO_2_/Al_2_O_3_ nanolaminate structure (SiO_2_‐to‐Al_2_O_3_ sublayer thickness ratio = 10:1).

Figure 4b shows optical simulation results obtained for n‐DBR structures with various material combinations using finite‐difference time‐domain, along with experimental results for n‐DBR4. Our group previously reported n‐DBR2s prepared using Al_2_O_3_ and TiO_2_ [45, 46]. However, the n‐DBR2s exhibit average high UV transmittances of 2.6%, and prolonged exposure to certain UV wavelengths severely degrades the outdoor reliability. For enhanced functionality, we construct nanolaminated LRI and HRI layers using only LRI and HRI materials, respectively. For the resultant n‐DBR4, the UV transmittance is reduced by 40% compared with n‐DBR2; however, the visible light transmittance is exceeded. The average visible‐ and UV‐light transmittances of the simulated n‐DBR4 are 94.15% and 1.4%, respectively, whereas those of the n‐DBR4 manufactured based on the simulation are 95.99% and 1.63%.

The n‐DBR UV‐blocking performance was evaluated using a UV aligner delivering 16.63‐mW cm^−^^2^ irradiation intensity. Figure 4c compares the UV‐shielding capabilities of thin films and substrates commonly employed in OLED fabrication. The UV transmittance values ​​of transparent PET and glass substrates were 82.74% and 92.42%, respectively, which shows that their UV blocking properties are quite poor. Similarly, LRI and HRI layers have exhibited UV transmittances of 91.88% and 76.72%, respectively; insufficient to ensure optical stability for organic devices. In contrast, the developed n‐DBR4 demonstrates outstanding UV‐blocking efficiency, with transmittance <0.5%. Under outdoor conditions with peak UV exposure, the UV intensity meter measures 16.63 mW cm^−^^2^, which drops to 0.42 mW cm^−^^2^ following passage through the n‐DBR filter—corresponding to a 97.47% reduction in UV intensity.

The impact of parylene‐C layers in the MFM structure on both the environmental and mechanical stability were investigated. The n‐DBR4‐ and MFM‐system barrier performance and mechanical robustness were assessed based on changes in the WVTR after cyclic bending and hygrothermal aging. Figure 4d reports the WVTRs of various barrier configurations measured at 30°C and 90% relative humidity (RH). For comparison, the HRI and LRI layers (60‐nm thickness) were fabricated via nanolaminate deposition at 40°C, achieving WVTRs on the order of 10^−^^4^ g m^−^^2^ day^−^^1^. The HRI layer, enriched in TiO_2_, yields 1.08 × 10^−^^4^ g m^−^^2^ day^−^^1^, benefiting from a multi‐interfacial, defect‐decoupling nanolaminate architecture and suppressed TiO_2_ and HfO_2_ crystallinity. The SiO_2_‐rich LRI layer exhibits a slightly higher WVTR of 3.43 × 10^−^^4^ g m^−^^2^ day^−^^1^. Therefore, average WVTRs of 2.79 × 10^−^^6^ and 1.263 × 10^−^^6^ g m^−^^2^ day^−^^1^, respectively, are obtained for the n‐DBR4 and parylene‐C/n‐DBR4/parylene‐C structures.

The mechanical flexibility was further evaluated via tensile testing, including a freestanding water‐surface tensile test, which provided substrate‐free measurements of the thin‐film fracture strain. Figure 4e shows the fracture elongations (ε) of the n‐DBR4, parylene‐C/n‐DBR4 (PN), and MFM systems. Symmetric multilayers with organic outer layers (SMOs) exhibit higher ε than asymmetric multilayer (AM) configurations. Surprisingly, the AM configuration fractures at the same strain as bare n‐DBR4; this is because the parylene‐C layer has insufficient fracture toughness to arrest crack propagation from the brittle n‐DBR4. In the SMO configuration, the surrounding parylene‐C layers distribute the strain energy bidirectionally, delaying catastrophic fracture. The single‐layer n‐DBR4 and parylene‐C films fracture at 0.43% ± 0.03% and 6.72% ± 0.42% strain, respectively, whereas elongations of 0.61% ± 0.02% and 0.42% ± 0.03% are observed for the n‐DBR4/parylene‐C and MFM, respectively. The symmetric MFM achieves a significantly higher elongation of 1.63% ± 0.39%, confirming that encapsulation of an inorganic film within flexible organic layers markedly enhances the mechanical‐stress resistance.

Bending tests corroborate these trends; after 1000 cycles, the n‐DBR4 WVTR increases by more than 10‐fold at 0.93% strain (Figure 4f). The MFM with symmetrical structure maintains stable WVTRs within one order of magnitude up to 0.93% strain, only increasing to 3.76 × 10^−^^5^ g m^−^^2^ day^−^^1^ at 1.87% strain. This improved mechanical endurance is attributed to (i) pronounced membrane‐force reduction due to stress compensation and (ii) mechanical buffering of the brittle n‐DBR4 by the softer parylene‐C layers.

The environmental durability was tested under damp‐heat conditions (85°C/85% RH) (Figure 4g). In general, ALD Al_2_O_3_ is known to be easily degraded by environmental conditions, and in our study, it lost its barrier properties due to rapid deterioration in just 20 h [31, 45, 47]. Although the n‐DBR4 comprises robust TiO_2_/HfO_2_ and Al_2_O_3_/SiO_2_ nanolaminates, it degrades steadily, reaching 7.63 × 10^−^^4^ g m^−^^2^ day^−^^1^ WVTR after 80 h. Atomic force microscope (AFM) analysis reveals no significant surface‐roughness change, suggesting environmental degradation driven by internal cracking from the high membrane force (Figure S7). In contrast, MFM multibarriers maintain WVTRs near 10^−^^6^ g m^−^^2^ day^−^^1^ for 60 h, which reaches only 2.54 × 10^−^^5^ g m^−^^2^ day^−^^1^ after 80 h. This value is approximately 30 times lower than that of the n‐DBR4. Therefore, stress‐compensating organic layers substantially improve the hygrothermal stability of brittle inorganic barrier films.

### 2.4 The Therapeutic Effect of SOLED Patch on Wound Healing

**FIGURE 5:**
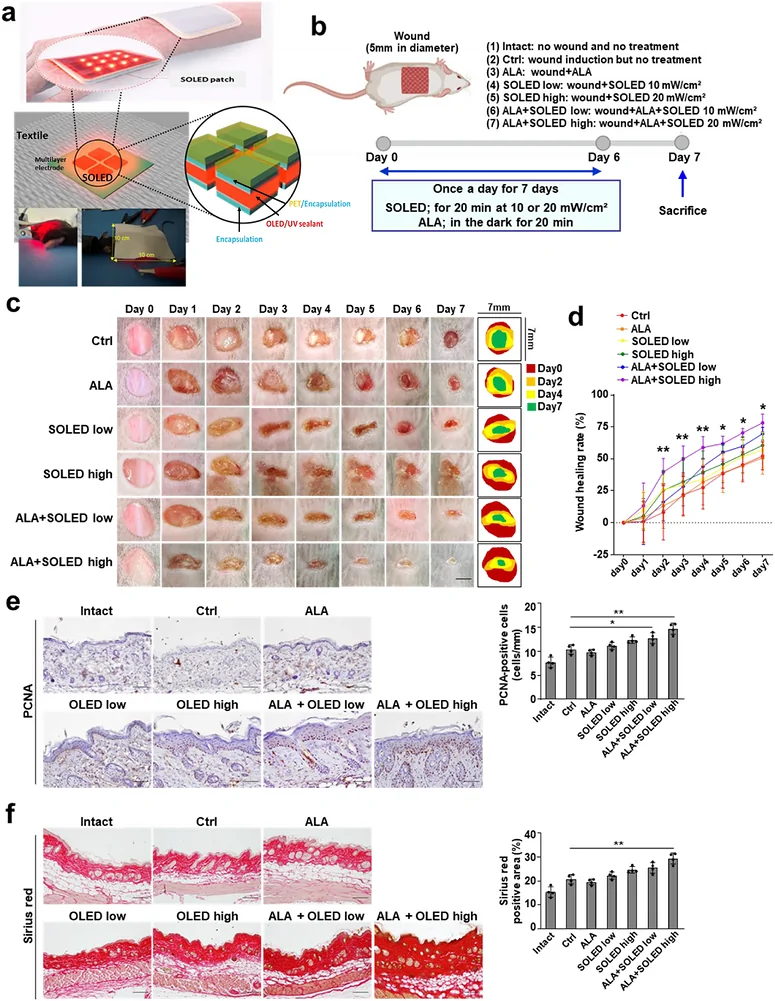
The SOLED treatment accelerates wound healing by promoting cell proliferation, angiogenesis, collagen deposition in vivo. (a) Schematic illustration and photographic demonstration of the biointegrated SOLED patch for phototherapeutic applications. (b) Schematic illustration of the experimental design. Full‐thickness excisional wounds (5 mm in diameter) were generated on the dorsal skin of mice, followed by daily ALA and SOLED treatment for 7 days. Mice were sacrificed on day 7 after wounding. (c) Representative macroscopic images of wound closure in each group from day 0 to day 7. Scale bar = 2.5 mm. (d) Quantification of wound healing rates over time, expressed as the percentage of wound closure relative to complete wound closure. (e) Immunohistochemical staining of PCNA in skin tissues and PCNA‐positive keratinocytes number analysis per epidermal‐layer length (mm). Scale bar = 50 µm. (f) Sirius red staining of wound tissues and Sirius red‐positive area analysis (%). Scale bar = 100 µm. Data are presented as mean ± SD. ^*^ *p* < 0.05, ^**^ *p* < 0.01 compared with the Ctrl group.

SOLED has emerged as a promising light source for PDT applications owing to its unique advantages, as it can be fabricated on ultrathin and soft substrates, enabling intimate skin contact and uniform light delivery to irregular or curved anatomical surfaces [48]. Figure 5a has been revised to clearly present the final textile‐based SOLED patch prototype used for the in vivo wound‐healing experiments. The prototype was fabricated on a 10 × 10 cm^2^ textile substrate and integrated four multilayer stretchable OLED units, enabling large‐area, deformable, and skin‐conformal light delivery. For the in vivo experiments, the textile‐based SOLED patch was operated at a power density of 20 mW cm^−^^2^, at which no apparent low‐temperature thermal damage was observed. Under these irradiation conditions, we evaluated the wound‐healing efficacy of SOLED‐based phototherapy, in which SOLED irradiation alone was used to examine red‐light‐induced therapeutic responses, while 5‐aminolevulinic acid (ALA)‐assisted SOLED irradiation was used to assess PDT‐mediated wound‐healing effects. The device was powered by a Keithley source meter through external wires connected to the OLED electrodes. Owing to its soft textile‐based patch configuration, the device could be placed in intimate contact with the murine skin during treatment, thereby enabling conformal optical delivery to the wound area. For effective PDT treatment using 5‐ALA as a photosensitizer, we fabricated VSOLED devices emitting red light. Encouraged by the wearable and biointegrated display technologies of the SOLED, we further verified the clinical application of our device using a wound healing model. A schematic illustration of a murine skin wound model and the treatment process is depicted in Figure 5b. Full‐thickness wounds were created on the backs of mice using a 5‐mm biopsy punch. Mice were randomly divided into seven groups: (1) Intact (no wound and no treatment), (2) Control (Ctrl, wound induction without treatment), (3) ALA (wound + ALA), (4) SOLED low (wound + SOLED irradiation at 10 mW/cm^2^), (5) SOLED high (wound + SOLED irradiation at 20 mW/cm^2^), (6) ALA + SOLED low (wound + ALA + SOLED irradiation at 10 mW/cm^2^), and (7) ALA + SOLED high (wound + ALA + SOLED irradiation at 20 mW/cm^2^). The wound areas were administrated with ALA in the dark for 20 min, followed by the SOLED irradiation (20 min). The wound closure of the mice in each group was monitored every day after the therapy, with complete wound closure defined as 100%, and the closure rate at each time point was calculated relative to the initial wound size on day 0 to analyze the treatment effect. As shown in Figure 5c,d, the ALA + SOLED high group exhibited a rapid wound‐healing trend compared to all experimental groups, reaching 78.21% ± 6.87% wound healing on day 7. In contrast, ALA, SOLED low, SOLED high, and ALA + SOLED low showed 50.71% ± 12.75%, 56.85% ± 13.96%, 60.6% ± 10.25%, and 69.66% ± 5.04% wound healing, respectively (Figure 5c,d), with no statistically significant differences compared to the control group throughout the healing process. These findings indicate that PDT treatment with our SOLED patch improves wound healing outcomes through faster and more effective wound closure.

The adequate vascularization is crucial for supplying oxygen and nutrients for repair, and cell proliferation is pivotal for wound closure. In addition, effective collagen deposition is vital for enhancing tissue tensile strength and promoting healing [44]. As illustrated in Figure 5e and Figure S8a, the number of proliferating cell nuclear antigen (PCNA)‐positive keratinocytes and blood vessels per field in the dermis was significantly higher in the ALA + SOLED high group compared with the control group. The ALA + SOLED high group also showed higher positive area of vascular endothelial growth factor A (VEGFA) and Sirius‐red than the control group (Figure 5f and Figure S8b). In contrast, no differences were observed among control, ALA, SOLED low, and SOLED high, although the ALA + SOLED low group revealed the higher number of PCNA‐positive keratinocytes and blood vessels in the dermis compared with the control group. These results suggest that PDT treatment based on SOLED increases cell proliferation, angiogenesis, and collagen deposition, which accelerates the wound‐healing process.

### 2.5 Potential Mechanisms Underlying SOLED Patch–Wound Healing

**FIGURE 6:**
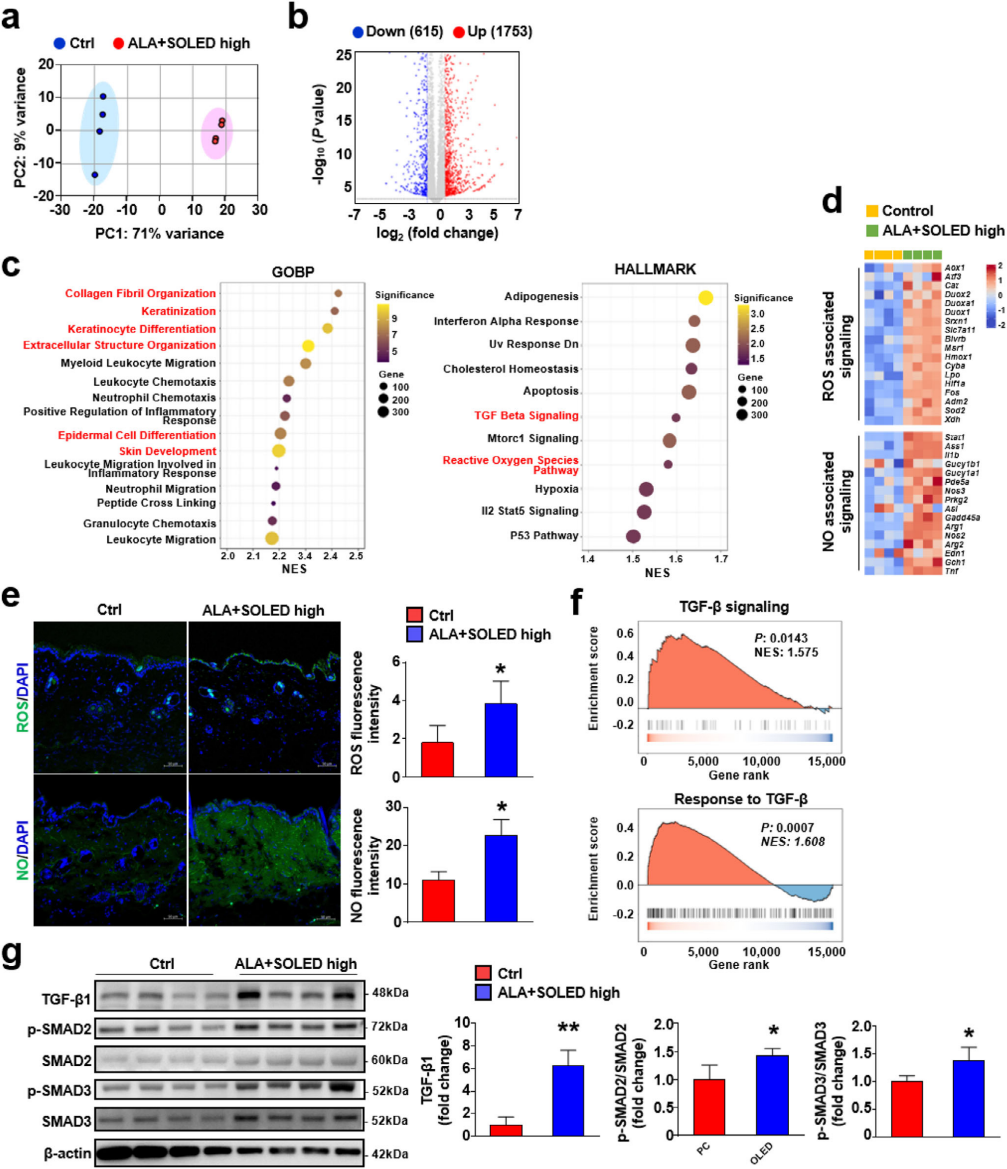
The SOLED treatment enhances RONS generation and activates TGF‐β signaling during wound healing in vivo. (a) Principal component analysis (PCA) plot showing transcriptomic separation between the Control and ALA + SOLED high groups. (b) Volcano plot showing differentially expressed genes (DEGs) between the two groups. Red and blue dots indicate significantly upregulated and downregulated genes, respectively. (c) Gene Ontology biological process (GOBP) and HALLMARK pathway enrichment analyses. (d) Heatmap analysis of ROS‐ and NO‐associated genes in the Control and ALA + SOLED high groups. (e) Representative fluorescence images and quantification of ROS and NO levels in skin tissues detected using DCFH‐DA and DAF‐FM DA staining on day 6, respectively. Scale bar = 100 µm. (f) Gene set enrichment analysis (GSEA) demonstrating enrichment of HALLMARK TGF‐β signaling and GO‐BP response to TGF‐β pathways in the ALA + SOLED high group. (g) Western blot analysis and quantification of TGF‐β1 and SMAD2/3 expression in skin tissues. Data are presented as mean ± SD. ^*^ *p* < 0.05, ^**^ *p* < 0.01 compared with the Ctrl group.

To systemically elucidate the molecular mechanisms underlying SOLED‐mediated wound healing, RNA‐seq based transcriptomic profiling was performed on skin tissues from the ALA + SOLED high and control groups. Principal component analysis (PCA) revealed distinct and highly reproducible clustering between the two groups, confirming that the ALA + SOLED high induces a fundamental shift in the cellular transcriptomic landscape (Figure 6a). Volcano plot analysis identified a significant number of differentially expressed genes (DEGs), with 1753 genes upregulated and 615 genes downregulated in the ALA + SOLED high group (Figure 6b). Gene Ontology biological process (GOBP) analysis indicated significant enrichment in keratinocyte differentiation, keratinization, keratinocyte differentiation, collagen fibril organization, extracellular structure organization, and skin development (Figure 6c). Gene set enrichment analysis (GSEA) and heatmap visualization further confirmed the robust upregulation of cell proliferation, angiogenesis, and fibrosis‐relative genes in the ALA + SOLED high group (Figure S9), consistent with the findings shown in Figure 5e,f and Figure S8. In addition, HALLMARK analysis revealed increased enrichment of reactive oxygen species (ROS) signaling pathways and TGF‐β signaling in the ALA + SOLED high group (Figure 6c). Heatmap analysis further demonstrated increased expression of ROS‐ and nitric oxide (NO)‐related genes in the ALA + SOLED high group (Figure 6d). PDT‐induced moderate ROS and NO generation can promote wound healing by enhancing epidermal stem cell differentiation, proliferation, and migration without significant cytotoxicity [44, 49, 50, 51]. Here, the ALA + SOLED high group exhibited markedly increased ROS and NO fluorescence intensities in both the epidermis and dermis, whereas minimal fluorescence signals were observed in the control group (Figure 6e). Because reactive oxygen and nitrogen species (RONS) are known to modulate TGF‐β‐related signaling pathways and fibrosis‐associated responses [52, 53, 54], we further investigated TGF‐β signaling activity in the wound tissues. GSEA demonstrated enrichment of TGF‐β signaling and response to TGF‐β pathways in the ALA + SOLED high group (Figure 6f). Consistently, Western blot analysis demonstrated increased expression of TGF‐β1 and activation of the SMAD2 and SMAD3 in the ALA + SOLED high group (Figure 6g). Collectively, these findings suggest that ALA + SOLED treatment enhances RONS generation and activation of the TGF‐β signaling pathway, potentially contributing to cell proliferation, angiogenesis, and collagen production during wound healing.

## 3 Conclusion

This study demonstrated a mechanically and environmentally reliable multilayer SOLED architecture that resolves the fundamental pixel density/ stretchability trade‐off. By vertically separating the emissive islands and interconnections, high‐FF pixelization and enhanced strain accommodation were achieved, as verified by simulations and experimental stretching tests. Integration of a parylene‐C/n‐DBR4/parylene‐C multifunctional encapsulation ensured optical stability and long‐term protection against water vapor, UV radiation, and hygrothermal degradation. Following prolonged UV irradiation and in accelerated damp‐heat testing, the encapsulated SOLEDs exhibited stable electroluminescent performance under 50% uniaxial strain, underscoring their robustness for practical applications. Overall, these advances establish a comprehensive strategy for realizing high‐resolution, mechanically robust, and environmentally durable SOLEDs that are compatible with scalable fabrication methods. The demonstrated architecture advances the development of future wearable and biointegrated display technologies, thereby expanding the design freedom and reliability of stretchable optoelectronics.

## 4 Experimental Section

### 4.1 Fabrication of Stretchable Patterned Metal Mask

The first‐layer electrode architecture was prepared using spin‐coating and photolithographic patterning to achieve high‐resolution, stretchable electrode features across a large area. The overall fabrication procedure consisted of four key stages: (1) preparation of the patterned metal mask, (2) SU‐8 patterning, (3) elastomer transfer, and (4) electrode deposition. Initially, a 100‐nm‐thick chromium (Cr) layer was deposited onto a glass substrate via vacuum thermal evaporation. A positive photoresist (AZ GXR‐601) was subsequently spin‐coated at 4000 rpm for 30 s, followed by soft baking at 130°C for 3 min. UV exposure was carried out through a photomask at an energy density of 17 mJ/cm^2^ for 6 s, and a post‐exposure bake was performed at 130°C for 10 min. After development using AZ 300 MIF developer and wet etching with Cr‐7 solution, the Cr layer was patterned into the desired geometry. The residual photoresist was removed with acetone, yielding a stretchable patterned Cr metal mask. The fabricated mask incorporated five patterned regions: first‐layer electrodes, first‐layer via holes, second‐layer electrodes, second‐layer via holes, and rigid island areas.

### 4.2 Fabrication of Stretchable Patterned Electrodes

A 5 wt.% polyvinyl alcohol (PVA) sacrificial layer was spin‐coated onto the Cr‐patterned glass mask at 1000 rpm for 50 s and baked at 130°C for 5 min. To define the mold structure, SU‐8 2010 was spin‐coated at 2000 rpm for 30 s, forming an approximately 20‐µm‐thick layer. A soft bake at 130°C for 2 min was conducted to remove residual solvent. UV exposure (17 mJ/cm^2^, 15 s) was then applied, followed by a post‐exposure bake at 130°C for 1 min. After development in SU‐8 developer, a patterned SU‐8 mold was obtained.

Ecoflex 00–20 elastomer was prepared by mixing the base and curing agent (Part A and Part B) in a 1:1 weight ratio. The mixture was spin‐coated onto the SU‐8 mold at 500 rpm for 10 s and allowed to cure at room temperature (25°C) for 2 h. The cured SU‐8/Ecoflex composite film was then gently released using deionized (DI) water. Subsequently, the first and second metal electrode layers, as well as the island structures, were deposited through a shadow mask using vacuum thermal evaporation under a base pressure of 10^−^^6^ Torr.

### 4.3 Fabrication of Stretchable Patterned via Holes

For via‐hole mold fabrication, SU‐8 was directly processed on the Cr‐patterned glass substrate without applying a PVA layer. To achieve a thickness of approximately 40 µm, the SU‐8 spin‐coating (2000 rpm, 30 s) and soft‐baking (130°C, 2 min) steps were repeated twice. UV exposure was conducted at 17 mJ/cm^2^ for 22 s, followed by development to form the via‐hole mold structures.

Next, a 5 wt.% PVA layer was spin‐coated onto the SU‐8 mold at 1000 rpm for 50 s and baked at 130°C for 5 min. Ecoflex (Part A:Part B = 1:1) was then spin‐coated at 2000 rpm for 40 s. Either the first‐ or second‐layer electrode was precisely aligned and transferred onto the elastomer surface, followed by natural curing at room temperature for 24 h. During curing, the electrode film was placed in a vacuum desiccator to gradually eliminate interfacial air bubbles. Finally, the entire structure was immersed in DI water to dissolve the sacrificial PVA layer and release the stretchable film. This sequential transfer process was repeated to construct a triple‐layer stretchable configuration incorporating the final island layer.

### 4.4 Fabrication and Characterization of VSOLED Devices

A semi‐transparent Ag layer was first deposited via thermal evaporation under a high vacuum of 10^−^^6^ Torr. A hole‐injection layer (HIL) of MoO_3_ (5 nm) and a hole‐transport layer (HTL) of N,N′‐bis(naphthalen‐1‐yl)‐N,N′‐bis(phenyl)‐benzidine (NPB, 67.5 nm) were sequentially deposited. The emitting layer (EML), consisting of bis(10‐hydroxybenzo[h]quinolinato)beryllium doped with 8 wt.% Ir(piq)_3_ in bebq_2_ (70 nm), was then thermally evaporated. Subsequently, Cs_2_CO_3_ (1 nm) was deposited as the electron‐injection layer (EIL), followed by deposition of a semi‐transparent Ag layer (30 nm) serving as the cathode for light emission. To complete the vertically stacked VSOLED structure, the second OLED unit was fabricated in reverse order on the semi‐transparent Ag layer. Cs_2_CO_3_ (1 nm) was first deposited as the EIL, followed by the same EML (bebq_2_:Ir(piq)_3_, 70 nm), NPB (67.5 nm) as the HTL, and MoO_3_ (5 nm) as the HIL. Finally, a 100 nm thick Al layer was deposited as the reflective electrode, thereby forming an efficient bottom‐emitting OLED structure.

### 4.5 Fabrication of n‐DBR and Parylene‐C for MFM

Al_2_O_3_, SiO_2_, HfO_2_, and TiO_2_ thin films were deposited at 70°C using a plasma‐enhanced atomic layer deposition system (Nexusbe, Plaminar). The metal precursors were as follows: trimethylaluminum for Al_2_O_3_, di‐isopropylamino silane for SiO_2_, tetrakis(cyclopentadienyl)hafnium(IV) for HfO_2_, and tetrakis(dimethylamido)titanium for TiO_2_. In all depositions, O_2_ plasma served as the oxidizing agent, which was used to improve the film quality and ensure complete removal of the precursor ligands. The n‐DBR4 structure was fabricated by stacking 6.5 pairs of nanolaminated HRI layers based on TiO_2_ and HfO_2_ and LRI layers based on Al_2_O_3_ and SiO_2_; the thicknesses of the first and last layers were adjusted to suppress transmittance ripples.

Parylene‐C was deposited by using a parylene coater (OBT‐PC300, Obang Technology). Parylene‐C powder was first vaporized at 80°C–175°C and then pyrolyzed into monomeric species at 690°C. The monomer was subsequently polymerized onto the surface of the substrate within the deposition chamber at room temperature to form a conformal parylene‐C coating.

### 4.6 Laser Cutting of Stretchable OLEDs

Encapsulated polyethylene terephthalate (PET) substrates were cut using a laser cutting system, and the laser path was programmed using a computer‐aided design program (Auto‐CAD, Autodesk Inc.). A CO_2_ laser with 10.6‐µm wavelength and 100‐W power was employed to cut the PET films into the desired shapes. The laser scanning speed was set to 290 mm/s and cutting was performed under ambient conditions. To ensure clean and precise cutting without thermal damage or deformation, the edges were inspected using an optical microscope.

### 4.7 Finite Element Method Simulation of Triple‐Layer Stretchable Structure

FEA was conducted using ANSYS Mechanical software to analyze the mechanical deformation behavior of the triple‐layer stretchable structure. The simulation quantitatively evaluated the strain distribution within the designed triple‐layer architecture. The Young's modulus and Poisson's ratio for the Ecoflex and SU‐8 were set to 0.5 MPa and 0.49, and 4 GPa and 0.22, respectively, based on values reported in the literature [11]. The thickness of each layer was defined based on the target dimensions described in the main text.

To ensure full mechanical coupling, the interfaces between the layers were modeled using bonded contact conditions. For the uniaxial tensile simulation, one edge of the model was fixed but the opposite edge was displaced to facilitate a natural Poisson contraction; this setup replicated the experimental boundary conditions. To facilitate in‐plane deformation along the tensile direction, the bottom surface of the 400‐µm‐thick Ecoflex layer was constrained with a frictionless condition. To evaluate the mechanical response of the stretchable structure, the von Mises equivalent strain values were extracted from the simulation results and analyzed under various applied strain levels.

### 4.8 Calcium Corrosion Test for WVTR Measurements

The WVTRs of the barrier samples were evaluated using an electrical calcium (Ca) test under controlled conditions of 30°C and 90% RH. The Ca sensor was fabricated through sequential thermal evaporation of patterned Al (100 nm) and Ca (250 nm) layers onto a glass substrate. Subsequently, the barrier‐coated PET film was laminated onto the Ca‐sensor cell using a UV‐curable sealant (XNR5570, Nagase).

### 4.9 Sample Preparation and Free‐Standing Tensile Test

Encapsulation barriers were initially deposited on copper (Cu)‐coated Si wafers. The barrier/Cu bilayer was then patterned into a dog‐bone geometry using a femtosecond laser system (PHAROS). The patterned films were floated on the water surface and subsequently transferred to a Cu etchant to remove the sacrificial Cu layer. Finally, the released barrier films were floated back onto water and subjected to tensile testing. A water surface, which had high surface tension (73 mN m^−^^1^) and low viscosity (1.002 mPa s), provided a stable platform for supporting the free‐standing thin films.

### 4.10 Surface Roughness

The surface morphologies of the thin films were characterized by atomic force microscopy (AFM; NX‐10, Park Systems) operated in non‐contact mode.

### 4.11 Optical Transmittance

The optical transmittance spectra were recorded using a UV–vis spectrophotometer (Cary 5000, Agilent) equipped with an integrating sphere.

### 4.12 Scanning Electron Microscopy

The surface morphologies of the polymer and barrier films were further examined using a field‐emission scanning electron microscope (FE‐SEM; Helios G4 CX, Thermo Scientific).

### 4.13 Animal Experiment

Six‐week‐old female C57BL/6 mice were randomly assigned to seven groups (*n* = 5 per group): (1) Intact: no wound and no treatment; (2) Ctrl: wound induction without treatment; (3) ALA: wound + ALA without SOLED irradiation; (4) SOLED low: wound + SOLED irradiation (10 mW/cm^2^); (5) SOLED high: wound + SOLED irradiation (20 mW/cm^2^); (6) ALA + SOLED low: wound + ALA + SOLED irradiation (10 mW/cm^2^); (7) ALA + SOLED high: wound + ALA + SOLED irradiation (20 mW/cm^2^). Full‐thickness excisional wounds (5 mm in diameter) were created on the shaved dorsal skin using a biopsy punch. Depending on the experimental group, wounds were treated with topical ALA (10% in saline, 20 µL per wound), SOLED irradiation, or a combination of both. For ALA‐treated groups, ALA was applied topically and incubated in the dark for 20 min prior to treatment. SOLED‐treated groups were subsequently exposed to SOLED irradiation once daily for seven consecutive days. On day 7 post‐wounding, mice were euthanized, and an 8‐mm skin area encompassing the wound margin was collected. All animal experiments were approved by the Institutional Animal Care and Use Committee of Chungnam National University (approval number: 202407A‐CNU‐147 and 202604A‐CNU‐041).

### 4.14 Histopathology and Immunohistochemistry

Skin tissues were fixed in 10% neutral‐buffered formalin, paraffin‐embedded, sectioned at 4 µm, and subjected to histological and immunohistochemical analyses. Collagen deposition was evaluated by Sirius red staining, and the positive area was quantified from three randomly selected fields per section using ImageJ. Blood vessels were counted in hematoxylin and eosin‐stained sections and expressed as vessel density per unit area (200x field). For immunohistochemistry, sections were stained with antibodies against VEGFA and PCNA, followed by hematoxylin counterstaining. VEGFA expression was quantified as the dermal‐positive area, while PCNA‐positive cells in the basal epidermal layer were counted and normalized to the analyzed length.

### 4.15 ROS and NO Detection

ROS and NO levels in mouse skin tissues were evaluated using DCFH‐DA and DAF‐FM DA on day6, respectively. Cryosectioned skin tissues were incubated with each probe (5 µm) for 1 h in the dark, washed with PBS, and analyzed by fluorescence microscopy under identical magnification conditions (200×). For fluorescence quantification, three randomly selected fields per mouse were analyzed, and the averaged value was used as a single biological replicate. ROS and NO quantification was performed using tissues from *n* = 4 and 5 mice respectively.

### 4.16 Western Blotting

Skin tissues were homogenized in RIPA lysis buffer containing protease inhibitors, and protein concentrations were quantified. Equal amounts of protein (30 µg) were separated by SDS‐PAGE and transferred onto PVDF membranes. After blocking with 5% skim milk in PBST, membranes were incubated overnight at 4°C with primary antibodies against TGF‐β1 (Abcam), SMAD2 (Cell Signaling Technology), SMAD3 (Santa Cruz Biotechnology), phospho‐SMAD2 (Cell Signaling Technology), phospho‐SMAD3 (Cell Signaling Technology), and β‐actin (Sigma–Aldrich). Membranes were then incubated with secondary antibodies, and protein bands were visualized using a luminograph (ATTO, Tokyo, Japan).

### 4.17 Transcriptomic Data Analysis

Total RNA was extracted from the homogenized skin wound tissues using TRIzol. Raw RNA‐seq read counts were analyzed in R (v4.4.2). Sample‐level structure was explored by dimensionality reduction using t‐distributed stochastic neighbor embedding (t‐SNE) applied to normalized expression values. DEGs between wounded controls and ALA + OLED high–treated samples (*n* = 4 per group) were assessed with DESeq2 (v1.46.0) using the Wald test. Gene set enrichment analysis (GSEA) was performed using gene‐level statistics from DESeq2 as the ranking metric. Enrichment was computed with fgsea (v1.28.0) using 100 000 permutations and gene set size constraints of minSize = 5and maxSize = 500. Gene sets were obtained from the Molecular Signatures Database (MSigDB v2025.1, Mus musculus), including Gene Ontology Biological Process and HALLMARK collections.

### 4.18 Statistical Analysis

Data are presented as the mean ± standard deviation (SD). Statistical analysis was performed using GraphPad Prism 6 software (GraphPad, CA, USA). For the analysis, two‐way ANOVA was followed by Tukey's post‐hoc tests in Figure 5d. Statistical significance among three or more groups was evaluated using one‐way ANOVA followed by Tukey's post‐hoc test, whereas comparisons between two groups were analyzed using an unpaired, two‐tailed Student's t‐test.

## Author Contributions

**Young Hyun Son**: conceptualization, methodology, software, data curation, investigation, writing – original draft. **Jeong Hyun Kwon**: project administration, supervision, conceptualization, methodology, data curation, writing – original draft, writing – review and editing. **Myeongheon Lee**: methodology, data curation, investigation. **Kyung Cheol Choi**: supervision, data curation, conceptualization, methodology, project administration, writing – review and editing. **Jun‐Yeop Song**: data curation. **Hyo‐Jung Kwon**: data curation, supervision, writing – review and editing.

## Conflicts of Interest

The authors declare no conflicts of interest.

## Supporting information




**Supporting File**: advs76176‐sup‐0001‐SuppMat.pdf.

## Data Availability

The data that supports the findings of this study are available in the supplementary material of this article and from the corresponding author upon reasonable request.
