# Supplementary material for: Biointegrated Multilayer Stretchable OLED Platform With Strain‐Decoupled Architecture for Durable Phototherapeutic Applications
Source: Adv Sci (Weinh). 2026 Jun 16:e76176. Online ahead of print. doi: 10.1002/advs.76176 (PMC13336443; doi:10.1002/advs.76176)
Supplement: Supplementary file 1 — Supporting File: advs76176‐sup‐0001‐SuppMat.pdf. [file ADVS-9999-e76176-s001.pdf]

Biointegrated Multilayer Stretchable OLED Platform with Strain-Decoupled Architecture for Durable Phototherapeutic Applications

Young Hyun Son[1], Myeongheon Lee[2], Jun-Yeop Song[3], Hyo-Jung Kwon[3,] Kyung Cheol Choi[1,*] and Jeong Hyun Kwon[2,*]*

[1]School of Electrical Engineering, Korea Advanced Institute of Science and Technology (KAIST), Daejeon 34141, Republic of Korea

[2]School of Semiconductor Engineering, Chungbuk National University, Cheongju, 28644 Chungcheongbuk-do, Republic of Korea

[3]College of Veterinary Medicine, Chungnam National University, Daejeon, 34134, Republic of Korea

*e-mail: love6539@cbnu.ac.kr (Jeong Hyun Kwon); kyungcc@kaist.ac.kr (Kyung Cheol Choi); hyojung@cnu.ac.kr (Hyo-Jung Kwon)

Keywords: Stretchable OLEDs, Multilayer architecture, Resolution–stretchability trade-off, Multifunctional encapsulation, Bio medical application

Reference	Structure strategy	Fill Factor	Maximum stretchability	Encapsulation	Bio application
Adv. Mater. Technol. 5,2000187(2020)	Stress buffer	25	40	X	X
Nano Lett. 20, 1526-1535 (2020)	Pillar Substrate	50	50	O	X
Mat. Horiz. 12, 7580(2025)	Rotation membrane	60	40	O	X
Nat. Commun. 15,2411(2024)	Hidden area	85	30	O	X
Nat Commun 15, 7802 (2024)),	3D height alternant	80	55	O	X
This work	Multi-layer	74	50	O	O

Table S1. Quantitative comparison of representative strain-engineered stretchable OLED platforms reported in previous studies and the proposed multilayer SOLED architecture.

The proposed multilayer SOLED platform was compared with representative previously reported stretchable OLED architectures in terms of structural strategy, fill factor (FF), stretchability, encapsulation capability, and application functionality. Unlike conventional coplanar strain-engineered structures primarily focused on mechanical deformation accommodation, the present work integrates vertically decoupled routing, multifunctional encapsulation, and wearable phototherapeutic operation within a single multilayer platform.

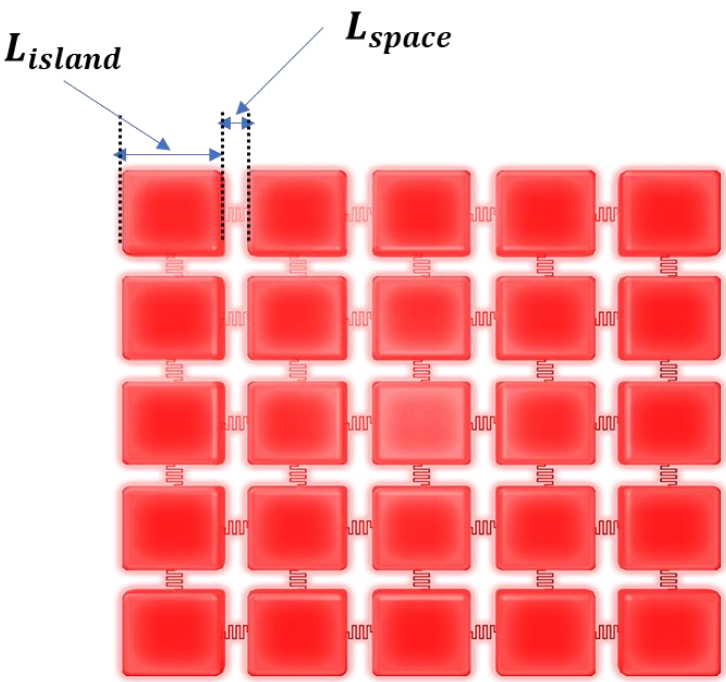

Figure S1. Fill factor (FF) calculation image for the multilayer SOLED architecture

The fill factor (FF) of the stretchable OLED platform was defined as the ratio of the emissive pixel area to the total device area:

Fill Factor (FF) = (Emissive area / Total device area) × 100 %

$$= (L_{island})^2 / (L_{island} + L_{space})^2 \text{ x } 100\ \%$$

For the proposed multilayer architecture, the vertically separated routing structure enabled the interconnect region to be positioned beneath the emissive layer, thereby minimizing the in-plane area occupied by the routing electrodes. In contrast, conventional coplanar island–bridge structures require additional lateral spacing for interconnect routing, resulting in reduced emissive area density.

The FF values were calculated based on the designed pixel geometry and layout dimensions shown in **Figure 1b**. Using this approach, the proposed multilayer structure achieved an FF of approximately 74.3%.

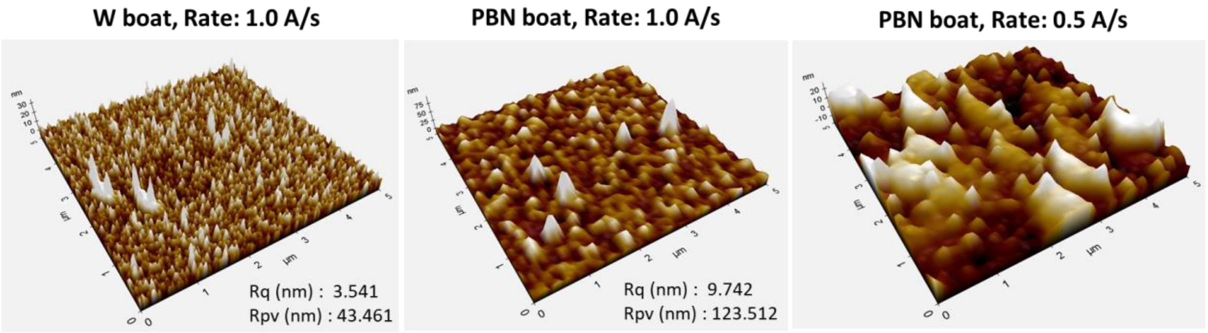

Figure S2. Surface roughness of 100 nm Al films deposited using different source heating boats and deposition rates

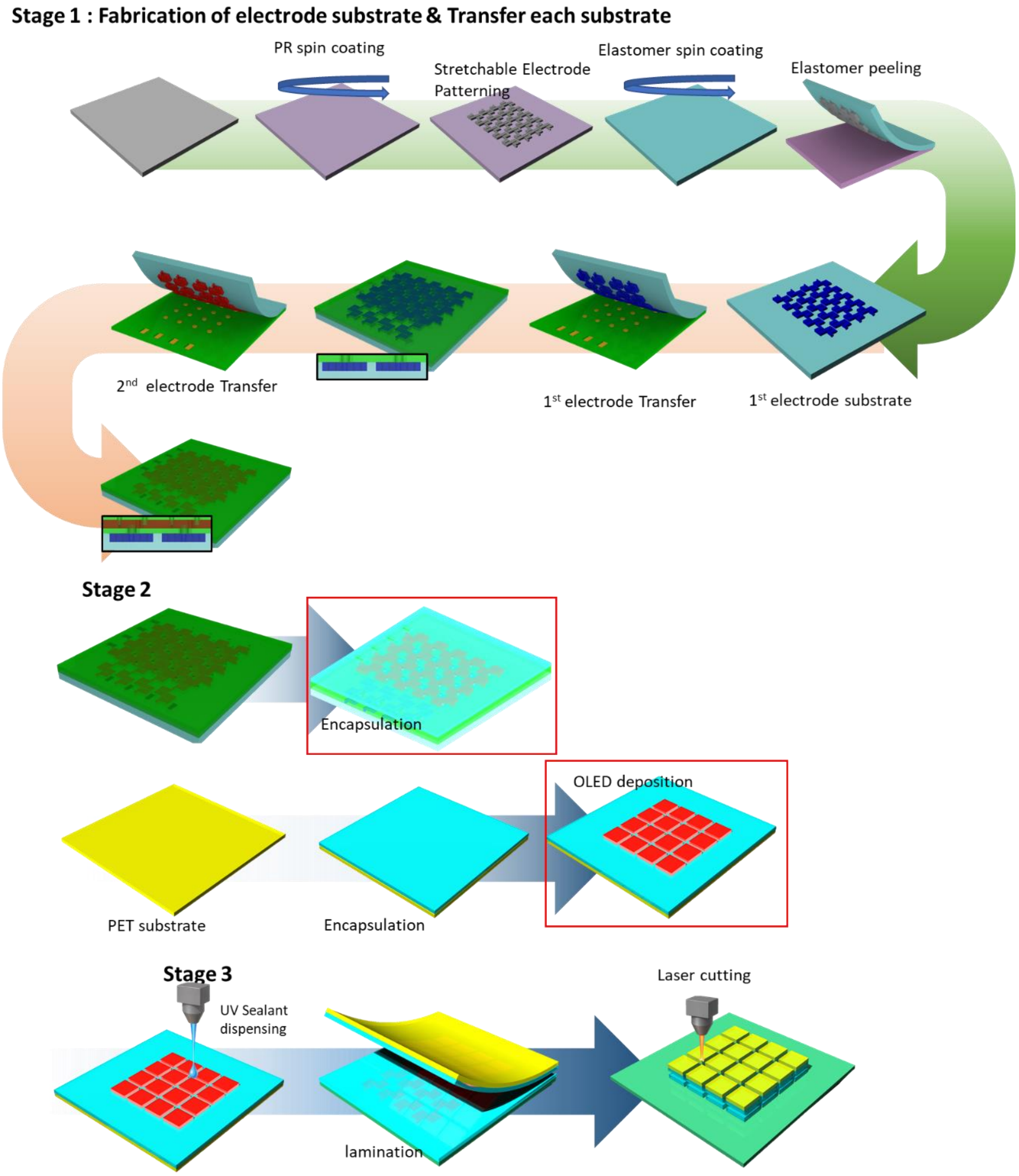

Figure S3. Detailed fabrication sequence of the PET-integrated multilayer stretchable OLED platform.

Stage 1: Preparation and transfer of stretchable electrode layers via photolithographic patterning and elastomer-assisted transfer.
Stage 2: Formation of the OLED device stack on a PET substrate, including bottom encapsulation, organic layer deposition, and top encapsulation to complete the OLED/encapsulation stack.

Stage 3: Deterministic lamination of the multilayer electrode substrate and OLED/encapsulation stack, followed by laser patterning to define the emission window and overall device geometry.

Stage 1.

Stretchable electrode layers were fabricated using standard photolithographic patterning. A photoresist (PR) layer was spin-coated onto a carrier substrate at 4000 rpm for 30s, followed by soft baking at 130°C. The electrode patterns were defined via UV exposure and development. Metal layers (Ag, 100 nm) were deposited using thermal evaporation/sputtering under a base pressure of 10^{-6} Torr.

Subsequently, an elastomer layer Ecoflex spin-coated at 1000 rpm was applied and cured at 30°C for 12 hours. The patterned metal electrodes were then transferred onto the elastomer substrate via controlled peeling, forming mechanically compliant first- and second-layer electrode networks.

Stage 2.

Thin-film encapsulation layers were simultaneously deposited on both bare PET and the stretchable electrode substrates. Subsequently, bottom-emitting OLEDs were fabricated on the encapsulated PET substrate.

Stage 3.

The encapsulated stretchable electrode substrate and the OLED/encapsulation stack were aligned using predefined alignment markers and laminated through a controlled transfer process.

Following lamination, the PET layer was laser-patterned using a CO_2 laser system operated at 100 W and $290mm\ s^{-1}$. The laser cutting process defined the emission window, sealing rim, and final device outline without damaging the active OLED region.

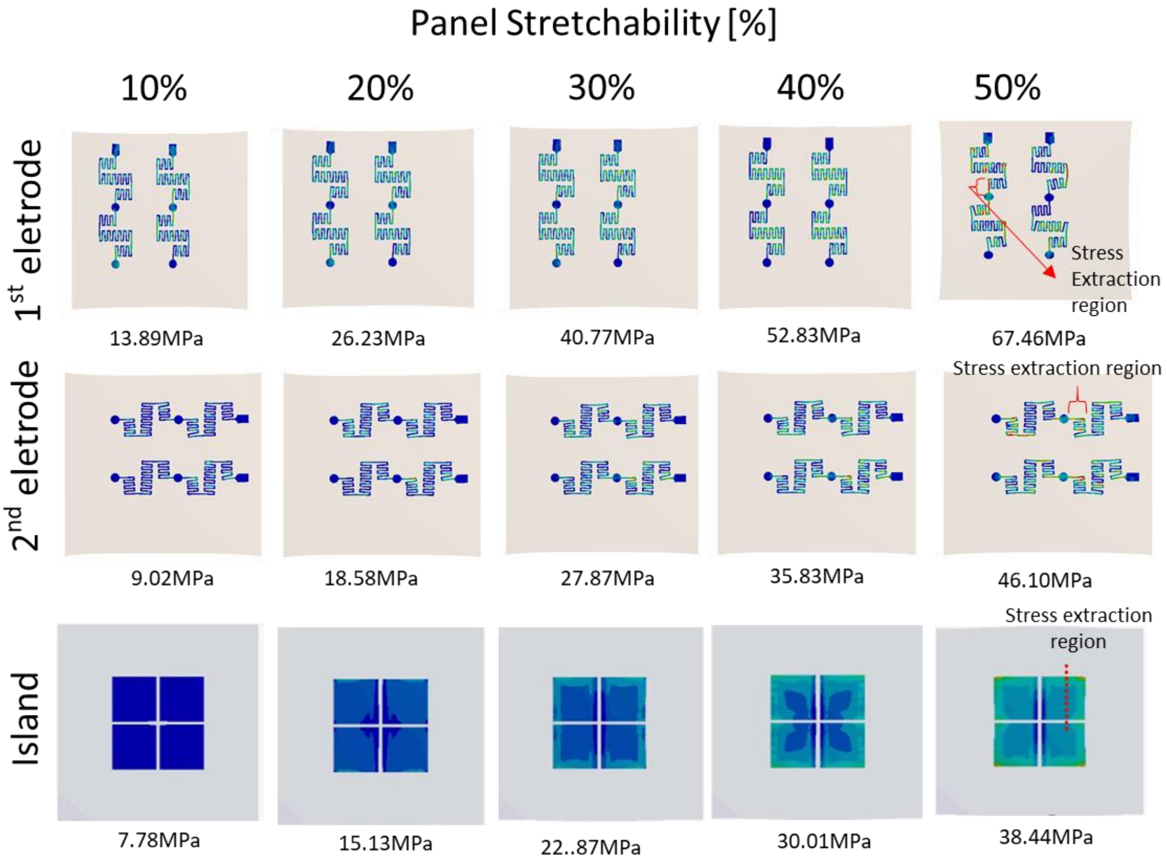

Figure S4. Simulated average stress distribution in the multilayer structure under increasing uniaxial tensile strain (10–50%). The calculated average stress values are shown for the first electrode layer, second electrode layer, and emissive island region. A progressive reduction in stress from the first electrode to the island region is observed at all applied strain levels, confirming effective stress redistribution and mechanical decoupling within the multilayer architecture.

Finite element simulations were performed under uniaxial tensile strain ranging from 10% to 50% to quantitatively evaluate stress redistribution within the multilayer structure. Average von Mises stress values were extracted from the highlighted representative regions of each functional layer: the first electrode layer (bottom routing layer), second electrode layer (intermediate routing layer), and emissive island region.

The multilayer structure was modeled using bonded contact conditions between adjacent layers to ensure full mechanical coupling throughout the integrated stack. The bottom surface of the elastomer substrate was assigned a frictionless support condition to allow in-plane deformation during tensile loading. For the uniaxial stretching simulation, one side of the panel was fixed, while tensile strain was applied by imposing displacement boundary conditions on the opposite side.

At all applied strain levels, the average stress decreased progressively from the first electrode layer to the emissive island region. For example, under 50% tensile strain, the average stress values were 67.46 MPa (first electrode), 46.10 MPa (second electrode), and 38.44 MPa (island), respectively. A similar trend was observed at lower strain levels (10–40%), indicating consistent stress attenuation through the vertically separated multilayer configuration.

This systematic reduction in stress across functional layers demonstrates that mechanical deformation is preferentially accommodated within the routing electrode layers, while the emissive island region experiences reduced mechanical loading. The results quantitatively support the strain-decoupling mechanism discussed in the main text and confirm that the multilayer architecture effectively mitigates stress concentration in the active OLED region under large tensile deformation.

[Boundary conditions used in the finite element simulations]

• Bonded contact conditions between adjacent layers

• Frictionless support condition applied to the bottom elastomer layer

• One edge fixed and the opposite edge displaced under uniaxial tensile loading

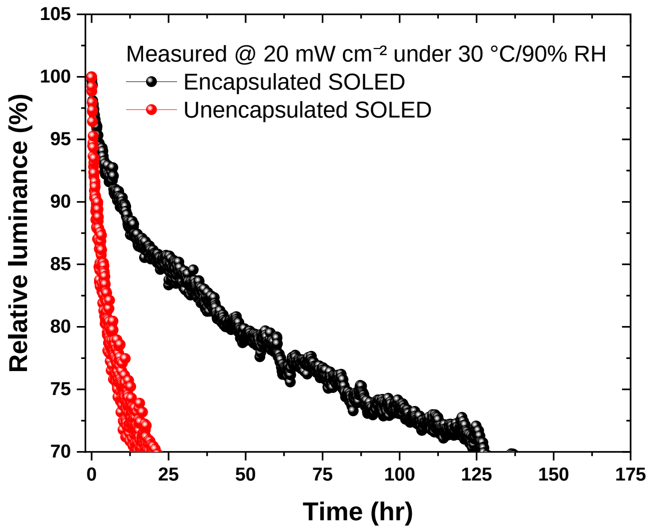

Figure S5. Accelerated lifetime of encapsulated and unencapsulated SOLEDs at 20 mW cm^{-2} under 30 °C/90% RH.

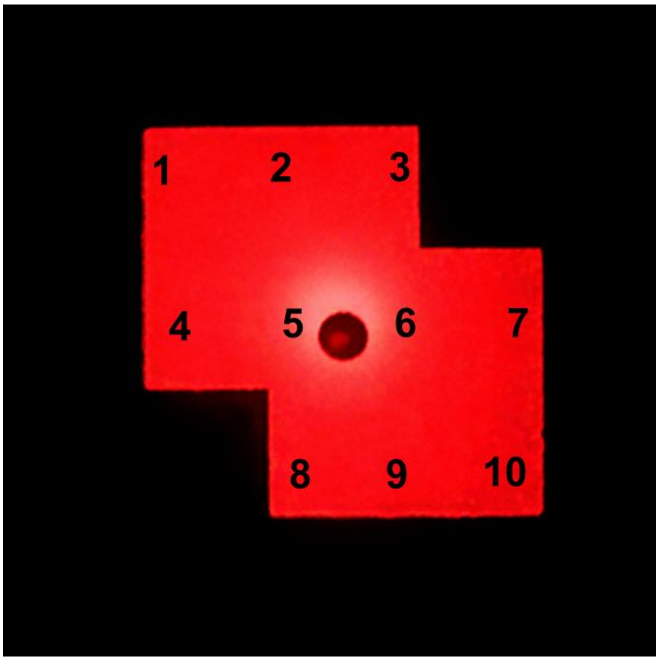

Figure S6. Numbered sampling positions across the emitting surface used for spatial irradiance mapping

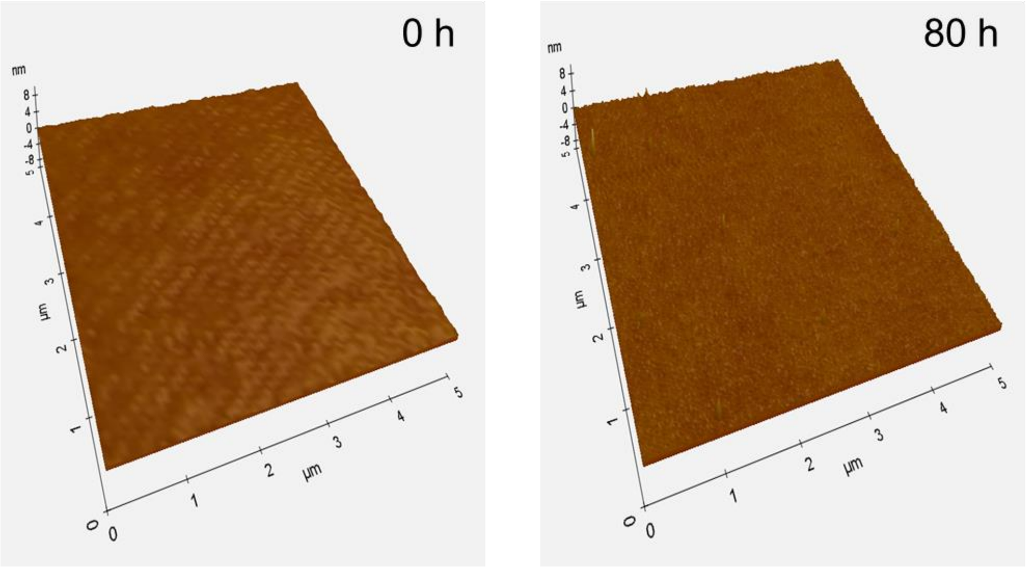

Figure S7. AFM surface morphology images of n-DBR4 before and after exposure to 85 °C/85% RH for 80 h.

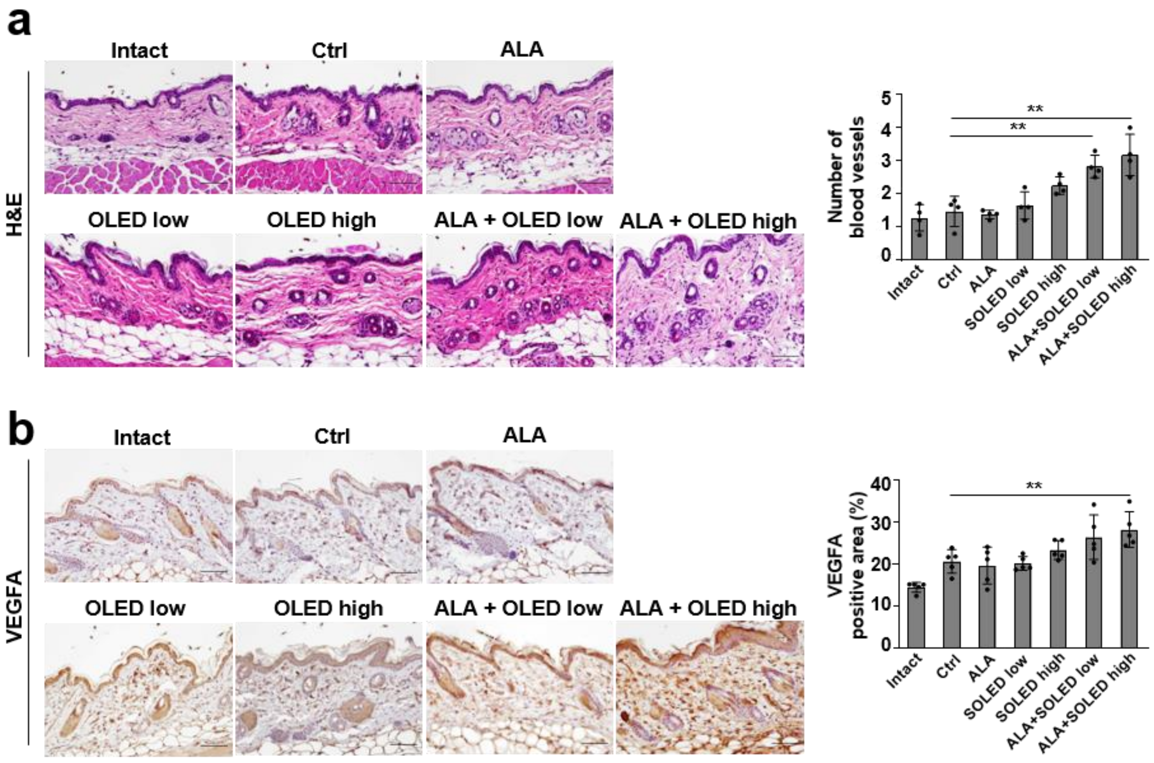

Figure S8. (a) Representative H&E staining, number of blood vessels in the dermis per unit area (mm^2). Scale bar = 50 μm. (b) VEGFA immunohistochemical staining, and VEGFA-positive area in dermal layer analysis (%). Scale bar = 50 μm. Data are presented as mean ± SD. $^{**}P < 0.01$.

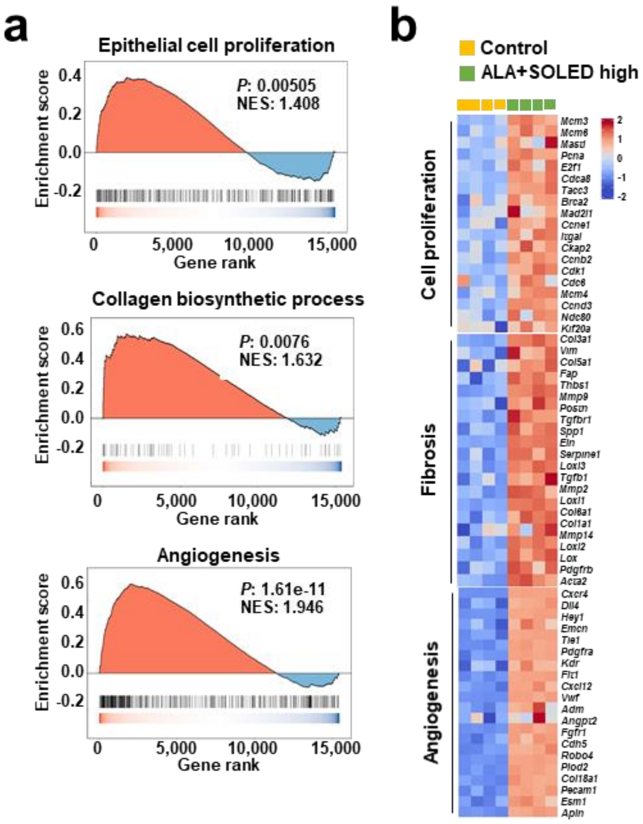

Figure S9. (a) Gene set enrichment analysis (GSEA) demonstrating enrichment of epithelial cell proliferation, collagen biosynthetic process, and angiogenesis in the ALA + SOLED high group. (b) Heatmap analysis of genes associated with cell proliferation, fibrosis, and angiogenesis in Control and ALA + SOLED high groups.
